# Pineal Region Glioblastoma, a Case Report and Literature Review

**DOI:** 10.3389/fonc.2017.00123

**Published:** 2017-06-12

**Authors:** Hayley Beacher Stowe, C. Ryan Miller, Jing Wu, Dina M. Randazzo, Andrew Wenhua Ju

**Affiliations:** ^1^Department of Radiation Oncology, Brody School of Medicine, Greenville, NC, United States; ^2^Department of Pathology, University of North Carolina School of Medicine, Chapel Hill, NC, United States; ^3^Center for Cancer Research National Cancer Institute, Bethesda, MD, United States; ^4^Department of Neuro-Oncology, Duke Health, Durham, NC, United States

**Keywords:** pineal, glioblastoma, radiotherapy, temozolomide, *O*-6-methylguanine-DNA methyltransferase, bevacizumab

## Abstract

**Introduction:**

Pineal region glioblastoma multiforme (GBM) is a rare disease entity with a generally poor prognosis. We present a case of a patient with an unresectable pineal region GBM treated with chemoradiation with favorable outcome.

**Case background:**

A 65-year-old patient who was presented with visual symptoms was found to have a pineal region tumor on imaging. A stereotactic biopsy showed a World Health Organization Grade IV GBM, *O*-6-methylguanine-DNA methyltransferase (MGMT) promoter methylated, isocitrate dehydrogenase 1 and 2 wild type. The patient was treated with radiotherapy with concurrent temozolomide, followed by adjuvant temozolomide. Disease progression occurred at 58 weeks post-biopsy, which prompted the initiation of bevacizumab. The patient was alive and functioning well as of his last follow up, 166 weeks from the initial biopsy.

**Discussion:**

On our review of the literature, 24 cases of pineal region GBM have been reported. The median reported survival for these previously reported cases was 6 months (range, 2–24 months). This patient has the longest overall survival reported to date for a patient with this diagnosis. This is the first patient in the literature with pineal region GBM who has been reported to have MGMT promoter methylation.

**Concluding remarks:**

Although pineal region GBM is a rare disease entity with a generally poor prognosis, long-term survival is achievable for select patients. MGMT promoter methylation may potentially have prognostic value. Favorable control of recurrent disease with the use of bevacizumab is possible.

## Introduction

Various tumor histologies can arise in the pineal region, including parenchymal tumors, neuroectodermal tumors, germ cell tumors, and meningeal tumors (1). Gliomas in the pineal region include fibrillary astrocytoma, pilocytic astrocytoma, anaplastic astrocytoma, glioblastoma, oligodendroglioma, ependymoma, and choroid plexus papilloma (2). Glioblastoma multiforme (GBM) is rarely found in the pineal region. This paper presents a case of primary GBM in the pineal region and discusses the clinical course, radiological findings, and treatment approaches with a review of the relevant literature. The patient provided written informed consent for his personal information to be used for research and publication.

## Case Background

**Figure 1:**
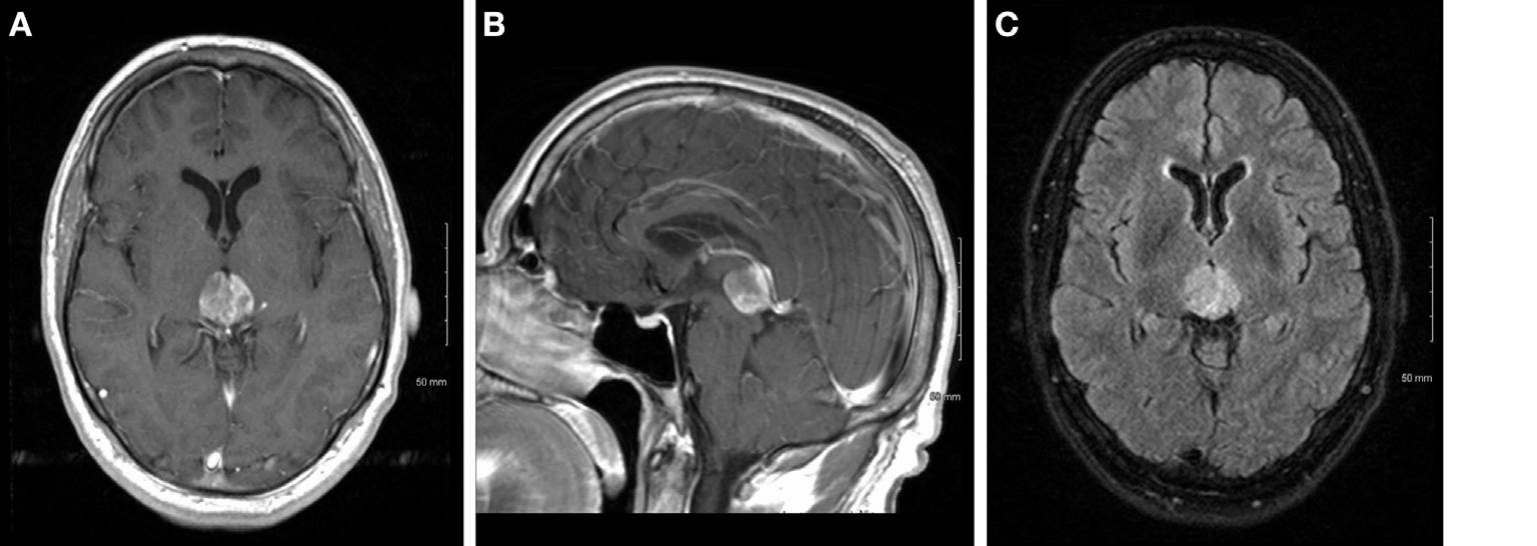
Axial **(A)** and sagittal **(B)** T1 post-contrast and T2 fluid attenuated inversion recovery **(C)** images of the tumor at initial presentation.

**Figure 2:**
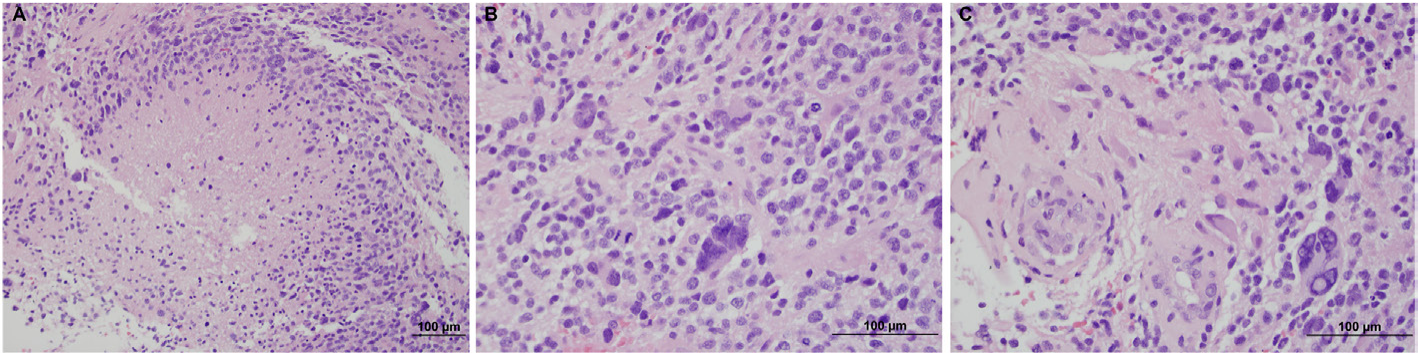
Hematoxylin and eosin stains of the tumor, showing necrosis at 20× **(A)**, mitotic figures at 40× **(B)**, and microvascular proliferation at 40× **(C)**.

**Figure 3:**
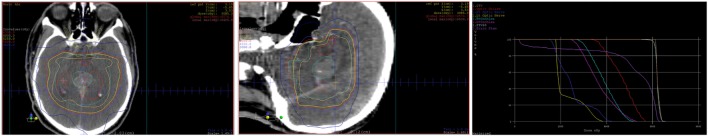
Axial and sagittal views of the dose distribution of the radiotherapy plan with the dose-volume histogram.

A 64-year-old male with no significant past medical history presented with vertical diplopia, headaches for 3 weeks, and 6 months of insomnia. His neurological examination revealed a right cranial nerve IV palsy and gait difficulties. Subsequent CT imaging, 1 month after initial presentation, revealed a poorly marginated, hyperdense mass located in the pineal region. A CT of the chest, abdomen, and pelvis, and an MRI of the total spine showed no evidence of metastatic disease. MRI of the brain revealed a heterogeneously enhancing pineal mass measuring 2.3 cm × 2.5 cm × 2.3 cm (Figure 1). The lesion was deemed unresectable due to its location and an image-guided stereotactic needle biopsy was performed. The pathology revealed a high-grade glioma composed of markedly atypical cells, many with giant nuclei and containing abundant mitotic activity (seven mitoses in three high-powered fields), multiple foci of microvascular proliferation, and areas of pseudopalisading necrosis, consistent with GBM, World Health Organization Grade IV (Figure 2). The tumor was positive for *O*-6-methylguanine-DNA methyltransferase (MGMT) methylation and negative for isocitrate dehydrogenase 1/2 mutations. The patient’s GBM was treated with concurrent chemoradiation followed by adjuvant chemotherapy (3, 4). The patient received intensity-modulated radiation therapy consisting of 60 Gy in 2 Gy daily fractions with concurrent temozolomide at 75 mg/m^2^. The initial fields were treated to a dose of 50 Gy and encompassed a 2 cm margin along white matter tracts from the enhancing tumor and the surrounding edema, and also covered the third and fourth ventricles, lateral ventricles, cerebral aqueduct, tectum, partial thalamus, and partial brainstem in the clinical target volume. The majority of the lateral ventricles were covered but the extreme ends of the horns were excluded due to concerns of additional toxicity, given the volume of brain treated, the prepontine cistern was not specifically targeted. An effort was made to cover the majority of the ventricles based on prior clinical reports describing a propensity for leptomeningeal spread of disease for pineal region GBMs. This initial radiotherapy plan was followed by a boost of 10 Gy to the enhancing tumor with a 2 cm margin along white matter tracts. The combined radiotherapy plan is displayed in Figure 3. The patient was placed on dexamethasone during the course of radiotherapy due to concerns that radiotherapy may potentially worsen local edema which could then result in obstruction of the cerebral aqueduct.

The patient developed alopecia and Common Terminology Criteria for Adverse Events version 4 Grade 1 dermatitis during the course of chemoradiation, but otherwise tolerated his concurrent chemoradiation well. Daily CT scans were used for radiotherapy image guidance and showed no signs of hydrocephalus developing during treatment. An MRI 4 weeks after completion of radiotherapy showed a slight decrease in the size of the pineal GBM to 2.3 cm × 2.0 cm × 1.9 cm. He continued on maintenance therapy with temozolomide, 5 days out of every 28 days per cycle, at 150 mg/m^2^ for the first cycle and increased to 200 mg/m^2^ for subsequent cycles.

**Figure 4:**
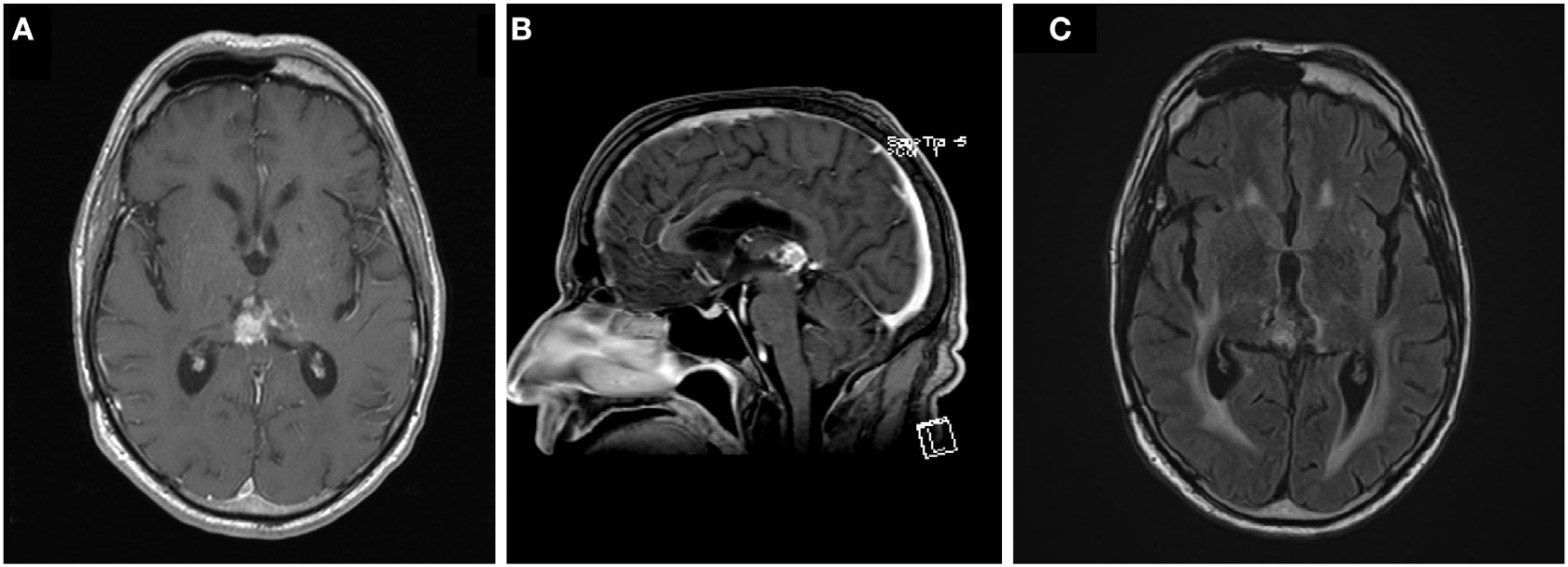
Axial **(A)** and sagittal **(B)** T1 post-contrast and T2 fluid attenuated inversion recovery **(C)** images of the tumor at last follow up, 166 weeks following the initiation of chemoradiation.

An MRI was performed at 58 weeks post-biopsy that showed two new lesions in the brainstem and parietal lobe, both within the prior radiotherapy fields. A total spine MRI did not reveal any drop metastases, nor was there any indication of CSF dissemination per lumbar puncture. Bevacizumab 7.5 mg/kg every 3 weeks was added to the temozolomide therapy (5). Temozolomide was discontinued after 17 cycles, a total of 88 weeks after the biopsy. Due to clinical symptoms of worsening gait disturbance, which corresponded to MRI findings suspicious for infarct within the radiotherapy fields, at 129 weeks post-biopsy, bevacizumab was discontinued and serial MRIs were subsequently performed every 2 months to closely monitor his disease. The MRI performed 146 weeks after the initial biopsy, when compared over multiple prior studies dating back to 111 weeks post-biopsy, showed subtle, progressive increase in size of the pineal mass from 11 mm × 11 mm to 14 mm × 11 mm and a confluent periventricular white matter T2/fluid attenuated inversion recovery hyperintensity that is non-specific and stable, this was redemonstrated in his MRI at 166 weeks after biopsy (Figure 4).

Clinically, the patient continues to have double vision, which is corrected by prism glasses. The diplopia continues to cause gait disturbance, which has improved with physical therapy and the patient no longer requires the use of a cane. He was last seen in clinic 166 weeks following his biopsy and 156 weeks following the initiation of chemoradiation for a follow up. He was continuously monitored closely off systemic therapy.

## Discussion

Tumors of the pineal region are uncommon intracranial neoplasms. The most common glioma in the pineal region is a well-differentiated astrocytoma (1). GBM, though common in the brain as a whole, is rarely seen in the pineal region. Since the first report on primary pineal GBM by Bradfield and Perez in 1972, 24 case reports have been described (6–23) (Table 1).

**Table 1:** Summary of published cases of pineal region glioblastoma.

Reference	Age	Sex	Symptoms	Radiographic findings	Leptomeningeal dissemination	Treatment	Survival
Bradfield et al. (6)	53	F	N/A	HCP, mass in posterior third ventricle	No (autopsy)	Resection	Post-operative death
Bradfield et al. (6)	5	F	N/A	HCP, mass in posterior third ventricle	No (autopsy)	Shunt	27 months
DeGirolami et al. (8)	3 cases		Increased ICP, vertical gaze palsy in one	N/A	N/A	RT for all three cases, resection for one	N/A
Kalyanaraman (12)	68	F	Ataxia, confusion, urinary incontinence, upgaze limitation	CT: HCP, calcified midline mass	N/A	Resection, RT	4 months
Norbut et al. (13)	36	F	HA, blurry vision, Parinaud’s Syndrome	CT: HCP, mass in posterior third ventricle	Yes	Shunt, RT	4 months
Frank et al. (10)	52	F	Intracranial ICP, oculomotor disturbances	HCP, mass in third ventricle	N/A	Stereotactic biopsy, RT	4 months
Edwards et al. (9)	12	F	N/A	N/A	N/A	Resection, RT, chemo	18 months
Vaquero et al. (17)	63	F	HA, behavioral changes	CT: rounded hyperdense, mass with ring enhancement	N/A	Shunt, resection, whole brain RT	6 months
Pople et al. (14)	6	F	HA, N/V, diplopia, decreased visual acuity, CN VI palsy, upgaze limited	CT and MRI: HCP, enhancing mass	Yes	Shunt, resection, local RT, chemo	4 months
Cho et al. (7)	63	M	Increased ICP, changing behavior	HCP, hyperdense pineal mass with enhancement	N/A	Resection, RT	6 months
Gasparetto et al. (11)	29	F	HA, drowsiness, fever, dizziness, seizure	CT and MRI: ill-defined heterogeneously enhanced mass with extension to thalamus	No	Shunt, resection	2 months
Toyooka et al. (15)	49	M	HA, diplopia, memory disturbance	MRI: HCP, irregular heterogeneously enhanced mass	Yes	Shunt, resection, chemo (ACNU), local RT	11 months
Amini et al. (16)	40	M	HA, N/V, diplopia, blurry vision	CT: obstructive HCP, strong enhancement, punctuate calcification MRI: heterogeneously enhancing with central necrosis, extension into midbrain	Yes	Endoscopic TVB, resection, shunt, whole brain RT, chemo (temozolomide)	5 months
Amini et al. (16)	43	M	Ha, disequilibrium, decreased level of mental status	MRI: heterogeneously enhancing, HCP	Yes	TVB, resection, whole brain RT, chemo	7 months
Amini et al. (16)	52	F	HA, N/V, diplopia, blurry gaze palsy	MRI: heterogeneously enhancing with central necrosis, obstructive HDC	Yes	Endoscopic TVB, RT	2 months
Moon et al. (20)	68	M	HA, N/V, ataxia	CT: HCP, hypodense mass MRI: irregular heterogeneously ring-enhanced mass with central necrosis	Yes	Resection, shunt	2 months
Ozgural et al. (18)	60	M	HA, ataxia	CT: triventricular HCP, isodense rounded mass MRI: heterogeneous, contrast enhanced mass	N/A	RT, chemo	24 months
Mansour et al. (21)	69	M	Altered mental status, vertigo	CT and MRI: heterogeneous mass, HCP	N/A	Biopsy, chemo, RT	16 months
Suzuki et al. (22)	65	M	Disturbed consciousness	CT: intraventricular hemorrhage, HCP	N/A	Resection, RT, chemo	N/A
Sugita et al. (23)	52	F	HA, memory disturbance	MRI: mass in pineal, HCP	N/A	Resection, RT, chemo	24 months
Sugita et al. (23)	18	M	HA, CN VI palsy	MRI: mass in the pineal, HCP	N/A	Resection, RT, chemo	13 months
Liu et al. (19)	30	M	HA, vomiting, LE numbness	MRI: multifocal lesion largest at the right thalamus	N/A	Resection, RT, chemo	N/A
Present case	65	M	Right CN IV palsy with gait difficulties	CT: heterogeneous mass	N/A	Biopsy, chemo, RT	>38 months

The clinical symptoms associated with the presentation of a pineal GBM are associated with compression of adjacent structures. Of the 24 patients described in literature, 66.7% presented with signs or symptoms of increased intracranial pressure, and 41.7% also presented with visual disturbances including diplopia, nystagmus, and blurry vision, which may be associated with Parinaud’s syndrome. CT scans of pineal GBM typically show heterogeneously contrast-enhancing masses with zones of low density. Pineal region GBM is shown to infiltrate the leptomeninges with 33.3% of patients in the literature.

Of the 24 cases in the literature, 54.1% have a survival duration of less than 11 months, and 70.8% having a survival duration of less than 24 months. Of the 18 patients where survival was reported (excluding our current patient), the median survival was 6 months (range, 2–24 months). Only one other patient has survived longer than 2 years. The maximum survival duration documented is the case presented in this report, with a survival time of 38 months at the time of manuscript preparation.

Pineal GBM is generally associated with a poor prognosis. Of the three cases that were treated with radiotherapy alone, the median survival was 3.5 months (range, 0–4 months) (10, 13, 16). The three patients in the literature treated with surgery alone had a median survival of 1.5 months, with a maximum survival of 2 months (6, 11, 20). Patients treated with resection followed by radiotherapy had a slightly higher median survival of 5.5 months (range, 4–6 months) (7, 12, 17). A tri-modal treatment approach of surgical resection followed by chemotherapy and radiotherapy yielded a median survival time of 12 months (range, 4–24 months) among the cases reported thus far in the literature (9, 14–16, 21, 23). However, those patients who received radiotherapy and chemotherapy without resection, including the present case, had the highest median survival duration of 20 months (range, 7–36 months) (16, 18, 21).

It is unknown why patients who did not undergo resection appear to have had a more favorable outcome in this review, indeed the difficulty of achieving a gross total resection in tumors located in this region is thought to be one of the factors that contribute to the poor prognosis of tumors in this region (7, 18, 20, 21), in addition to the propensity for pineal region GBMs to exhibit leptomeningeal spread of disease (13, 16, 21, 24), and the fact that progressive disease in this region could quickly affect critical structures in proximity to the pineal gland (16, 24). It may be possible that any attempt at resection could increase the risk of leptomeningeal seeding, and perhaps coverage of a significant volume of the ventricles in the radiotherapy plan as was done in this patient could aid in sterilizing leptomeningeal disease, but these hypotheses need to be studied in more detail.

*O*-6-methylguanine-DNA methyltransferase promoter methylation predicts for better outcomes for patients with GBM in other locations in the brain, including patients with an unresectable GBM (24). Our patient’s favorable outcome is likely associated with his MGMT promoter methylation status. To our knowledge, this is the first reported pineal GBM with MGMT promoter methylation. In prior literature, the methylation status was either not reported or was negative. A comparison of survival times of patients treated with chemoradiation based on MGMT promotor status would be valuable once more cases are reported, the results may lend more support to the premise that reasonable survival outcomes can be achieved with chemoradiation in MGMT promoter methylated patients in the absence of gross total resection.

Based on the literature review performed for this report, there is no reported utilization of bevacizumab in patients with pineal region GBM. Our patient had an excellent response to bevacizumab with good long-term control of disease after initial progression, which has contributed to his good overall survival. This favorable response reflects data seen in cases of recurrent GBM outside of the pineal region (5).

## Concluding Remarks

Pineal GBM is a rare disease that is associated with a poor prognosis. The majority of patients present with signs of increased intracranial pressure and visual symptoms. It is difficult to draw conclusions from the small number of total patients reported over a number of decades with differing treatment paradigms. This is the first pineal region GBM that reports positive MGMT promoter methylation and use of bevacizumab upon progression. Our case suggests that durable control of the disease can potentially be achieved in the absence of a gross total surgical resection. Further research in the role of MGMT methylation as a prognostic indicator and of the response of pineal region GBM to bevacizumab is warranted.

## Ethics Statement

The patient described in this case report consented to have his case described in this manuscript. Written permission was obtained from the patient’s treating medical oncologist for publication. The patient’s personal identifiers were not included in this manuscript.

## Author Contributions

HS: primary author of manuscript. CM: provided comments regarding case pathology with figures. JW: provided comments regarding patient’s systemic therapy and associated toxicities. DR: provided comments regarding patient’s systemic therapy and associated toxicities, provided radiographs. AJ: assisted primary author in manuscript preparation. Provided information regarding patient’s radiotherapy.

## Conflict of Interest Statement

The authors declare that the research was conducted in the absence of any commercial or financial relationships that could be construed as a potential conflict of interest.

## Abbreviations

GBM, glioblastoma multiforme; IMRT, intensity-modulated radiation therapy; MGMT, *O*-6-methylguanine-DNA methyltransferase; IDH, isocitrate dehydrogenase, WHO, World Health Organization.
